# First Clinical Results of Novel Haemodynamic Simulation Software for Patient-Specific Qp:Qs Quantification in Patients with Atrial Septal Defect Using Routine 2D Echocardiographic Data

**DOI:** 10.3390/jcm15093540

**Published:** 2026-05-06

**Authors:** Florian Gross, Robert Dragendorf, Teresa Lerach, Alexandra Hinke, Felix Berger, Raphael Seiler, Stanislav Ovrutskiy

**Affiliations:** 1 Department of Congenital Heart Disease—Pediatric Cardiology, Deutsches Herzzentrum der Charité, Augustenburger Platz 1, 13353 Berlin, Germany; 2 Charité—Universitätsmedizin Berlin, Corporate Member of Freie Universität Berlin and Humboldt-Universität zu Berlin, Charitéplatz 1, 10117 Berlin, Germany; 3 DZHK (German Centre for Cardiovascular Research), Partner Site Berlin, 10785 Berlin, Germany

**Keywords:** atrial septal defect, Qp:Qs, shunt quantification, echocardiography, haemodynamic simulation, digital twin modelling, congenital heart disease, non-invasive haemodynamics, computational cardiology, intracardiac shunt

## Abstract

**Background/Objectives:** Accurate quantification of left-to-right shunt volume is central to clinical decision-making in patients with atrial septal defect (ASD). Conventional echocardiographic Qp:Qs estimation is widely used but limited by operator dependency, Doppler alignment sensitivity, and the quadratic amplification of diameter measurement errors in flow calculations. These factors contribute to clinically relevant variability, particularly in paediatric populations with small vessel dimensions. Simulation-based haemodynamic modelling offers an alternative approach by integrating structural and functional cardiac parameters within a patient-specific computational framework, independent of direct Doppler flow measurements. **Methods**: This retrospective single-centre study evaluated agreement between conventional Doppler-derived and software-based Qp:Qs quantification using a three-dimensional haemodynamic simulation model. Transthoracic echocardiographic datasets from patients with isolated secundum ASD undergoing defect closure between 2018 and 2024 were analysed. Conventional Qp:Qs was calculated using pulmonary and aortic valve diameter and velocity–time integral measurements, while software-derived Qp:Qs was computed from patient-specific haemodynamic modelling based on cardiac chamber geometry and physiological parameters. Mean values were compared using Student’s *t*-test. Agreement was assessed using Bland–Altman analysis. A predefined ± 20% deviation threshold was considered clinically acceptable according to the study protocol. **Results**: A total of 98 echocardiographic examinations from 94 patients were included. Mean Qp:Qs was 1.83 ± 0.45 by echocardiography and 1.73 ± 0.47 by simulation modelling, with no statistically significant difference between methods (*p* Using the predefined ± 20% deviation criterion, 53.1% of examinations fell within the acceptable range. Larger ASD diameter was associated with increased inter-method deviation. **Conclusions**: This study provides an initial feasibility evaluation of a novel haemodynamic simulation approach enabling patient-specific three-dimensional modelling of cardiac structures and shunt physiology based on routine echocardiographic data. Simulation-derived Qp:Qs estimates demonstrated no systematic difference in comparison with conventional Doppler-based quantification and were feasible within routine clinical workflows. Ongoing prospective validation against invasive haemodynamic reference standards will further define analytical accuracy and potential clinical applicability.

## 1. Introduction

### 1.1. Clinical Relevance of Shunt Quantification in Atrial Septal Defect

Secundum atrial septal defect (ASD) is among the most common congenital heart defects and is frequently associated with chronic left-to-right shunting, resulting in right heart volume overload and long-term morbidity if left untreated [1]. Over time, this may lead to right atrial and right ventricular dilation and can be associated with complications such as tricuspid regurgitation, atrial arrhythmias, and pulmonary hypertension [2,3]. Early detection and precise quantification of the shunt are essential for therapeutic decision-making [3]. Central to the assessment of haemodynamic relevance and indication for defect closure is the quantification of the pulmonary-to-systemic blood flow ratio (Qp:Qs). A Qp:Qs ≥ 1.5 is widely accepted as a threshold indicating haemodynamically significant shunting and is incorporated into current treatment guidelines [2,4].

### 1.2. Echocardiographic Assessment of Qp:Qs and Methodological Limitations

Transthoracic echocardiography (TTE) is the established first-line non-invasive modality for the diagnosis, anatomical assessment, and follow-up of ASDs [3]. It is widely available, cost effective, and provides high-quality imaging without radiation exposure [5]. Beyond morphological characterisation, TTE allows non-invasive estimation of Qp:Qs using Doppler-derived velocity time integrals (VTI) and valve diameters [6].

However, Doppler-based shunt quantification is inherently dependent on image quality and measurement precision. Because vessel diameters are squared in flow calculations, even minor measurement inaccuracies may lead to substantial volumetric misestimation, with reported deviations of up to 25% [7]. Guidelines recommend measuring both diameter and Doppler signal at identical anatomical levels to ensure accuracy [8,9,10,11]. In clinical practice, stroke volume can be derived from either the left ventricular outflow tract or the aortic valve plane; in the present study, measurements were performed at valve level to ensure reproducible anatomical referencing. Averaging multiple cardiac cycles further improves measurement reliability [8].

Alternative approaches to shunt quantification include invasive oximetry during cardiac catheterisation and phase-contrast cardiovascular magnetic resonance imaging (cMRI). Invasive measurement remains the physiological reference standard but is reserved for selected patients due to procedural risk and its invasive nature [2]. In addition, invasive haemodynamic assessment may be influenced by examination-related factors, including sedation or anaesthesia, patient positioning, ventilation conditions, and periprocedural haemodynamic alterations affecting systemic and pulmonary vascular resistance. These factors may contribute to measurement variability and deviations from resting physiological conditions [2]. cMRI provides high diagnostic accuracy but is not routinely required in patients with isolated secundum ASD [2,12].

### 1.3. Computational Haemodynamic Modelling and Digital Twin Concepts

Efforts to improve haemodynamic assessment have led to the development of computational cardiac models capable of simulating intracardiac blood flow. Some simulation tools have been applied in complex congenital heart disease, such as single-ventricle physiology, but often require invasive haemodynamic data for model calibration [13].

Other modelling frameworks rely on cross-sectional imaging modalities such as computed tomography (CT) or cMRI and are primarily designed to reproduce physiological anatomy and function rather than pathological haemodynamics in routine clinical settings [14,15,16]. As a result, translation of computational heart modelling into everyday echocardiographic workflows has remained limited.

The concept of the “Digital Twin” has emerged as a central vision of precision cardiology, aiming to create patient-specific, multi-scale cardiac models integrating anatomical, physiological, and haemodynamic information for diagnostic and therapeutic support [17]. Recent advances focus on automated image recognition and three-dimensional model generation derived from two-dimensional imaging datasets [18].

To our knowledge, no currently available software has been specifically validated for the quantification of Qp:Qs based solely on routine two-dimensional echocardiographic data.

### 1.4. Study Objective

The NovaHeart software (Version 1.1.5, AIBODY.IO Limited, London, UK) applies digital twin principles by generating a patient-specific haemodynamic model based exclusively on routine two-dimensional transthoracic echocardiographic measurements. By integrating structural geometry and physiological parameters into a computational framework, the platform enables automated simulation of intracardiac flow dynamics, including Qp:Qs.

Given the absence of clinically validated haemodynamic simulation tools derived from routine echocardiographic datasets, systematic evaluation of analytical performance is required prior to clinical implementation.

The primary aim of this study was therefore to compare Qp:Qs values obtained by conventional Doppler echocardiography with those derived from simulation-based haemodynamic modelling in patients with isolated secundum ASD, focusing on inter-method agreement and clinical applicability in a real-world imaging setting.

## 2. Materials and Methods

### 2.1. Study Design

This single-centre study analysed retrospective echocardiographic data to evaluate the novel three-dimensional echocardiography-based haemodynamic simulation software NovaHeart for the assessment of anatomical and haemodynamic characteristics in patients with ASD, with a specific focus on shunt quantification (Qp:Qs). This manuscript reports the retrospective method comparison between echocardiography-derived Qp:Qs, calculated using standard Doppler-based flow measurements, and software-derived Qp:Qs, estimated from echocardiographic measurements of cardiac chamber dimensions, defect geometry, and global haemodynamic parameters, without relying on Doppler flow measurements at the ventricular outflow tracts.

### 2.2. Ethics Approval

The study was reviewed and approved by the responsible ethics committee of the Charité—Universitätsmedizin Berlin (EA2/315/23, approved 17 June 2024). All data were handled in accordance with applicable data protection regulations (General Data Protection Regulation, GDPR and local regulations). Data were collected and stored in a pseudonymised REDCap database (Research Electronic Data Capture, Version 15.2.1); results are reported anonymously.

### 2.3. Patient Selection

We screened patients evaluated at the German Heart Centre in Berlin—Deutsches Herzzentrum der Charité (DHZC) for the presence of an ASD and the availability of routine transthoracic echocardiography (TTE) data of sufficient image quality suitable for Qp:Qs estimation and software-based modelling. The retrospective cohort comprised consecutive patients within the predefined study period (2018–2024) who met the predefined eligibility criteria.

### 2.4. Inclusion Criteria

Patients were eligible if they had a diagnosis of secundum ASD with haemodynamic relevance as defined by current guideline recommendations and available TTE data suitable for both conventional Qp:Qs calculation and software-based modelling. Patients of age groups 0–70 years were eligible.

Indication for defect closure was derived from echocardiographic assessment (TTE and/or intraprocedural transoesophageal echocardiography, TEE) according to current guidelines [2,19]. Cardiac catheterisation was performed primarily without fluoroscopy under TEE guidance [20]; no standardised invasive haemodynamic assessment including oximetry was performed.

### 2.5. Exclusion Criteria

Exclusion criteria comprised:

1. Insufficient data quality, including incomplete echocardiographic measurements, inadequate image quality, or missing key parameters required for either method;
2. Presence of any congenital heart disease other than isolated secundum ASD;
3. Pulmonary arterial hypertension;
4. Software-derived Qp:Qs values outside the physiologically plausible range (<1.0 or >3.0).

### 2.6. Echocardiographic Qp:Qs Assessment

Transthoracic echocardiographic examinations were performed using modern ultrasound systems (Vivid™ E90, GE Healthcare, Chicago, IL, USA). For this study, relevant parameters were retrospectively re-measured by a single investigator to ensure methodological consistency and minimise inter-observer variability [21]. All DICOM datasets were analysed using EchoPAC software (GE Healthcare, Chicago, IL, USA, Version 204).

Measurements followed a predefined protocol based on current guideline recommendations [3,5,22]. Comprehensive echocardiographic assessment included chamber quantification, M-mode, and Doppler measurements. Qp:Qs was calculated using standard Doppler echocardiography by comparing pulmonary (Qp) and systemic (Qs) blood flow. Pulmonary flow was derived from pulmonary valve diameter and pulsed-wave Doppler VTI measured at valve level, typically obtained from the parasternal short-axis view [6]. Systemic flow was calculated analogously using aortic valve diameter and pulsed-wave Doppler VTI measured at the aortic valve level from the parasternal long-axis view (PLAX) in accordance with current echocardiographic guideline recommendations [3].

Doppler measurements were performed with careful alignment of the Doppler beam to blood flow direction and were averaged over multiple cardiac cycles in accordance with guideline recommendations to reduce beat-to-beat variability [8,9].

### 2.7. Software-Based Qp:Qs Calculation

For software-based analysis, echocardiographic measurements were imported into the NovaHeart software (Version 1.1.5, AIBODY.IO Limited, London, UK). The software generates a patient-specific three-dimensional haemodynamic cardiac model designed to simulate intracardiac blood flow dynamics in patients with atrial septal defect.

**Figure 1:**
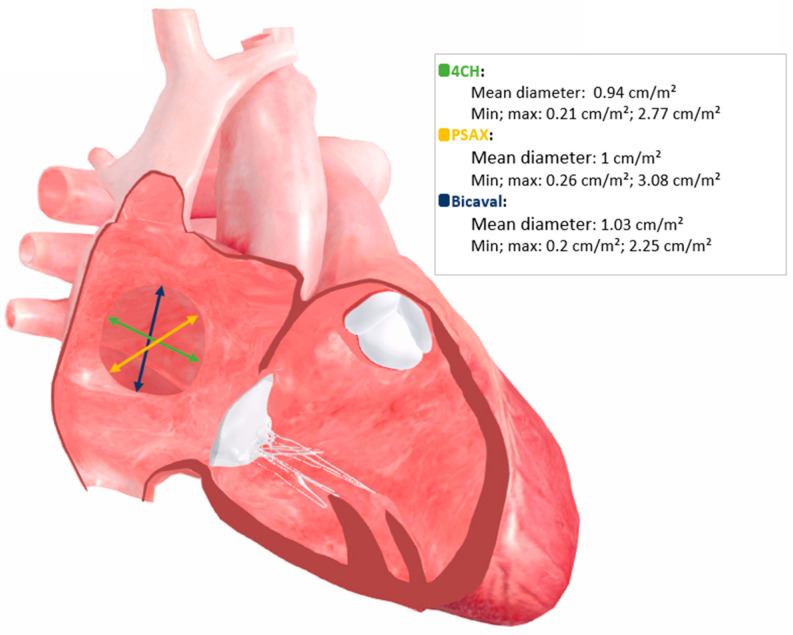
Patient-specific haemodynamic model reconstruction based on echocardiographic defect geometry.

Patient-specific simulations are generated from anatomical and haemodynamic parameters derived from routine transthoracic echocardiography, including chamber volumes and dimensions, ventricular end-diastolic volumes, and ASD size and spatial geometry. Based on these inputs, the software reconstructs a digital four-chamber haemodynamic model and automatically computes pulmonary (Qp) and systemic (Qs) blood flow, Qp:Qs ratio, and absolute shunt volume per cardiac cycle. An illustrative example of defect geometry integration into the haemodynamic simulation model is shown in Figure 1.

The model is based on a multi-chamber flow framework simulating pressure-driven blood movement between cardiac compartments. Blood flow across the interatrial communication is calculated using fundamental fluid dynamic principles. Continuity relationships for incompressible flow and Bernoulli-based pressure–velocity interactions are integrated, with additional consideration of viscous flow effects relative to defect size. Input parameters comprise haemodynamic chamber parameters, including left and right atrial volumes, ventricular end-diastolic volumes, and atrial and ventricular dimensions, as well as spatial ASD parameters, including defect diameter measured across the standardised echocardiographic planes (four-chamber view, bicaval view, parasternal short-axis view) and corresponding rim distances to the superior and inferior vena cava, aortic root, atrial roof, and crux cordis. The model outputs pulmonary and systemic blood flow, the Qp:Qs ratio, and absolute shunt volume per cardiac cycle.

In contrast to conventional Doppler-based calculations, the software does not rely on velocity time integrals or outflow tract flow measurements, but derives haemodynamic parameters from simulated intracardiac flow behaviour. Processing required only seconds per case and was compatible with routine clinical workflows.

### 2.8. Statistical Analysis

Statistical analyses were performed using IBM SPSS Statistics (Version 30.0, IBM Corp., Armonk, NY, USA). Continuous variables are presented as mean ± standard deviation or median with range, depending on distribution. Categorical variables are expressed as frequencies and percentages.

Normality of distribution was assessed using skewness and kurtosis evaluation. Both echocardiography-derived and software-derived Qp:Qs values demonstrated approximate normal distribution (skewness and kurtosis *z*-scores within ±2), supporting the use of parametric testing. Mean Qp:Qs values derived from conventional echocardiography and software-based modelling were compared using paired *t*-tests. Agreement between both methods was primarily assessed using Bland–Altman analysis, including calculation of mean bias and 95% limits of agreement (mean ± 1.96 standard deviations).

A predefined deviation threshold of ± 20% between software-derived and echocardiography-derived Qp:Qs values was considered clinically acceptable in accordance with the study protocol. This threshold was defined considering the known measurement variability of Doppler-derived Qp:Qs quantification, which may reach up to 25% due to quadratic error amplification in flow calculations [7], and reported mean overestimation of approximately 21% relative to invasive haemodynamic assessment [8]. The proportion of examinations falling within this predefined agreement range was calculated descriptively.

Effect size was quantified using Cohen’s *d* and interpreted according to established guidelines [23,24]. Scatter plots were generated to visualise inter-method relationships.

To explore potential determinants of inter-method variability, linear regression analyses were performed. Variables tested included ASD diameter, body surface area, age, and echocardiographic image quality.

All statistical tests were two-sided, and a *p*-value < 0.05 was considered statistically significant.

## 3. Results

### 3.1. Study Cohort

**Figure 2:**
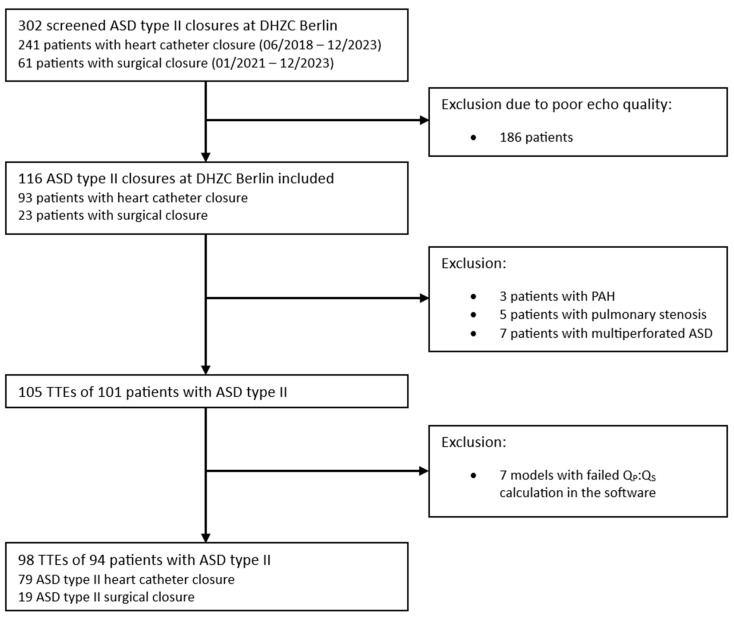
Study cohort selection flow chart.

A total of 98 transthoracic echocardiographic examinations from 94 patients with isolated secundum ASD met the predefined inclusion criteria. Examinations were performed within the study period from 2018 to 2024. The study inclusion process is summarised in Figure 2.

In cases where more than one echocardiographic examination per patient was available, additional datasets were included if they represented ambulatory follow-up assessments performed at least six months apart and fulfilled identical image quality and measurement criteria as baseline examinations. These repeated examinations were analysed independently, reflecting distinct haemodynamic assessment time points.

The gender distribution reflected known epidemiological trends, with 65% female patients (*n* = 64). The median age at examination was 5.98 years (range 0.6–69.9 years), and the median body weight was 22.9 kg (range 5–100 kg), representing a broad paediatric and adult congenital cohort. A detailed overview is shown in Table 1.

Across the cohort, Qp:Qs values derived from conventional echocardiography covered a wide haemodynamic spectrum, reflecting varying degrees of left-to-right shunting. This range enabled comparison of modelling performance across both moderate and haemodynamically significant defects.

Overall echocardiographic image quality was rated high in 87% of examinations, ensuring reliable measurement conditions. In the remaining studies, image quality was at least sufficient in all segments relevant for haemodynamic analysis.

### 3.2. Comparison of Qp:Qs Measurements

Mean Qp:Qs measured by conventional transthoracic echocardiography was 1.83 ± 0.45, compared with 1.73 ± 0.47 using the NovaHeart software. The mean inter-method difference (bias) was 0.10 ± 0.51, with a 95% confidence interval of −0.01 to 0.20.

Paired comparison of both methods demonstrated no statistically significant difference (*p* = 0.064). Effect size analysis revealed a negligible difference between measurement approaches, with Cohen’s *d* = 0.189 (95% CI −0.011 to 0.388). Pearson correlation analysis demonstrated a positive association between both methods (*r* = 0.387, *p* < 0.001).

Across the full measurement range, software-derived Qp:Qs values followed the distribution pattern observed in Doppler-derived calculations, and no clustering of deviations was observed at specific haemodynamic ranges.

**Figure 3:**
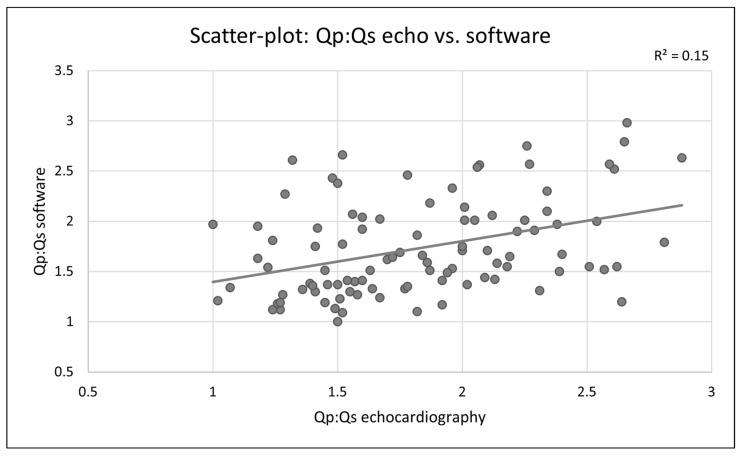
Scatter plot comparing echocardiography-derived and simulation-based Qp:Qs values.

Scatter plot analysis demonstrated a positive linear relationship between both measurement approaches. The correlation between both measurement approaches is illustrated in Figure 3.

### 3.3. Agreement Analysis

**Figure 4:**
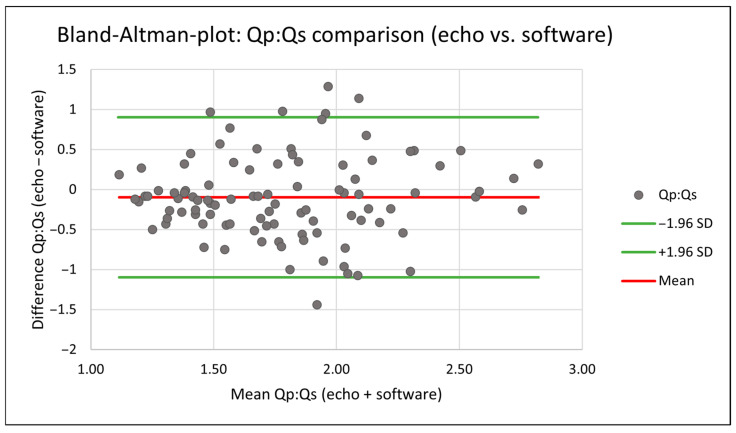
Bland–Altman analysis of echocardiography-derived versus simulation-based Qp:Qs measurements.

Agreement between both methods was further evaluated using Bland–Altman analysis. Bland–Altman analysis showed a mean bias of 0.10 and 95% limits of agreement from −0.90 to 1.10, with no evidence of proportional bias (Figure 4).

Absolute inter-method differences were symmetrically distributed around the mean bias, with no evidence of directional measurement drift. Furthermore, visual inspection did not suggest progressive widening of deviation across increasing shunt magnitudes.

### 3.4. Predefined Deviation Threshold

Using the predefined deviation threshold of ± 20% between software-derived and echocardiography-derived Qp:Qs values, 53.1% of examinations fell within the acceptable agreement range.

Examinations exceeding this deviation threshold were distributed across the full haemodynamic spectrum and were not confined to a single shunt severity category.

### 3.5. Influencing Factors

To explore potential determinants of inter-method variability, linear regression analyses were performed assessing the association between patient- and defect-related parameters and the absolute difference between echocardiography-derived and software-derived Qp:Qs values.

In linear regression analysis, ASD diameter emerged as a significant predictor of increasing inter-method discrepancy (*p* < 0.05). Larger defect sizes were associated with greater deviations between measurement approaches.

In contrast, patient age, body surface area, and echocardiographic image quality did not demonstrate statistically significant associations with inter-method differences. Regression modelling did not identify additional independent predictors of variability within the analysed parameter set.

## 4. Discussion

The present study evaluated agreement between conventional echocardiography-derived and software-based Qp:Qs quantification in patients with atrial septal defect using a patient-specific three-dimensional haemodynamic simulation model. This retrospective single-centre analysis represents an initial feasibility evaluation of digital haemodynamic modelling for non-invasive shunt quantification.

The primary objective was to determine whether software-derived Qp:Qs measurements demonstrate acceptable agreement with established Doppler-based calculations under routine clinical conditions and to characterise inter-method variability and determinants of measurement differences.

### 4.1. Measurement Comparison and Agreement

Comparison of Qp:Qs values derived from conventional echocardiography and software-based modelling demonstrated comparable mean values between both approaches. Mean values did not differ significantly, and inter-method bias was small, indicating the absence of systematic over- or underestimation by the simulation model.

Bland–Altman analysis further supported agreement across the measured range, with the majority of measurements falling within the 95% limits of agreement, and no proportional bias was identified. At a population level, simulation-based haemodynamic modelling therefore provided Qp:Qs estimates comparable to established echocardiographic calculations.

### 4.2. Agreement Relative to the Predefined Deviation Threshold

When applying the predefined agreement threshold of ± 20%, 53.1% of examinations fell within the acceptable range, and the primary protocol-defined endpoint was therefore not fully met.

This finding must be interpreted within the retrospective design and early validation phase of the modelling approach. Prospective validation incorporating invasive haemodynamic measurements is ongoing and will further clarify absolute model accuracy and calibration performance.

### 4.3. Methodological Considerations and Echocardiographic Limitations

Interpretation of inter-method variability must consider intrinsic limitations of conventional Doppler-derived Qp:Qs estimation. Accurate flow calculation depends on precise valve diameter acquisition and optimal Doppler beam alignment, with even minor measurement errors leading to substantial volumetric misestimation due to the quadratic relationship between diameter and flow. The relative percentage error associated with small absolute measurement deviations increases as vessel size decreases, rendering this limitation particularly relevant in paediatric cardiology and underscoring the need for measurement approaches less dependent on geometric precision.

Beyond systematic measurement uncertainty, Doppler-based quantification is also susceptible to outlier values arising from suboptimal acoustic windows, respiratory motion, and alignment variability. Such measurement outliers may disproportionately influence inter-method comparisons and contribute to observed deviations when echocardiography is used as the comparative reference modality [7].

Velocity time integral acquisition is additionally influenced by insonation angle, respiratory variability, and haemodynamic fluctuations. Furthermore, conventional calculations assume circular valve geometry and laminar flow, which may not fully reflect complex intracardiac shunt dynamics. Observed deviations should therefore be viewed as reflecting combined variability of two haemodynamic quantification approaches rather than modelling inaccuracy alone.

Previous comparative investigations have demonstrated that Doppler-derived Qp:Qs quantification exhibits variability when referenced against invasive haemodynamic assessment methods. In a retrospective analysis by Faherty et al., transthoracic echocardiography-derived Qp:Qs showed only limited correlation with oximetry-based measurements obtained during cardiac catheterisation (*R*^2^ = 0.32) and wide limits of agreement on Bland–Altman analysis, with a tendency toward overestimation of shunt magnitude, particularly in haemodynamically significant defects [8]. Complementary evidence from phase-contrast cMRI investigations further underscores modality-dependent differences in non-invasive shunt quantification when compared with invasive flow assessment [12]. Collectively, these findings highlight that Doppler-based shunt assessment itself is subject to methodological uncertainty and should therefore not be regarded as an error-free comparator in method comparison studies.

Despite these limitations, echocardiographic Qp:Qs was selected as the comparative reference for this initial validation for several reasons. It represents the most widely used non-invasive method for shunt quantification in clinical practice, is recommended by current guidelines as a validated haemodynamic assessment tool [2,19], and directly informs therapeutic decision-making. Furthermore, alternative non-invasive modalities such as cMRI are not routinely required in patients with isolated secundum ASD [2] and were therefore not available as a comparator across the full cohort. Agreement with this established clinical benchmark therefore constitutes a clinically meaningful first step prior to definitive validation against invasive haemodynamic measurements.

### 4.4. Impact of Defect Size on Modelling Accuracy

Defect size emerged as a significant determinant of inter-method variability, with larger ASDs associated with increasing deviation between measurement approaches. This observation is physiologically plausible, as shunt volume increases non-linearly with defect size and depends on complex interactions between atrial pressure gradients, ventricular compliance, and pulmonary vascular resistance.

In larger defects, small variations in geometric input parameters or haemodynamic assumptions may translate into greater simulated flow differences, whereas smaller defects demonstrated closer inter-method concordance. Patient age, body surface area, and echocardiographic image quality did not significantly influence agreement, suggesting modelling robustness across a broad congenital population.

### 4.5. Clinical Applicability and Workflow Implications

Beyond analytical agreement, simulation-based haemodynamic modelling offers potential clinical and workflow advantages. Conventional Doppler-derived Qp:Qs estimation is operator dependent and sensitive to acquisition technique, insonation angle, and measurement site.

By integrating structural and haemodynamic parameters within a unified computational framework, simulation modelling may reduce reliance on precise outflow tract alignment and repeated Doppler acquisitions. Automated processing enables standardised shunt quantification and reproducible estimates across operators, with processing times compatible with routine clinical workflows.

In addition to Qp:Qs estimation, modelling provides complementary parameters such as simulated flow distribution and absolute shunt volume, offering extended insight into haemodynamic burden.

### 4.6. Prospective Validation and Future Directions

Prospective validation against established invasive haemodynamic assessment methods represents an essential next step in the comprehensive evaluation of simulation-based shunt quantification. While the present study assessed agreement between two echocardiography-derived approaches, integration of catheter-based flow measurements will allow refinement of model calibration.

Future research should aim to validate simulation-based shunt quantification against invasive haemodynamic reference standards across varying shunt severities, refine physiological parameter integration, and define clinical applicability. Beyond validation, potential applications may include longitudinal follow-up, post-interventional remodelling assessment, and expansion to additional congenital shunt lesions. An ongoing prospective study at our centre is designed to address these objectives.

In parallel to the ongoing prospective validation against invasive haemodynamic reference standards, additional modelling functionalities are being explored within the research framework. Preliminary work has focused on the non-invasive estimation of right ventricular volumes derived from echocardiographic datasets, which is currently under investigation. The stepwise expansion and validation of haemodynamic simulation modules aim to further refine physiological model fidelity. In the future, integration of volumetric parameters into multimodal simulation environments may enhance shunt quantification by complementing flow-based metrics with chamber volume dynamics and ventricular loading conditions [25].

### 4.7. Study Limitations

Several limitations of the present study should be acknowledged. First, this analysis represents a retrospective single-centre investigation, which may limit generalisability across institutions, imaging platforms, and acquisition protocols. Although examinations were performed according to standardised institutional procedures, inter-centre variability in echocardiographic acquisition and measurement practices cannot be excluded.

Second, the study population consisted exclusively of patients with isolated secundum ASD undergoing defect closure. While this reflects a clinically relevant cohort, extrapolation to patients with multiple defects, other atrial-level shunt morphologies, or more complex congenital heart disease may be limited.

The study cohort was subject to selection bias toward haemodynamically significant defects, as the primary indication for defect closure had been established prior to referral to our tertiary interventional centre, and echocardiographic data used for the present analysis were obtained during the admission workup.

Furthermore, simulation outputs are inherently dependent on the quality and completeness of echocardiographic input parameters. Although only examinations with sufficient image quality were included, residual measurement variability in chamber dimensions or defect geometry may have influenced modelling results. Additionally, the analysis included 98 examinations from 94 patients, with 4 patients contributing repeated measurements analysed independently. While the small number of non-independent observations is unlikely to have materially influenced the results, this should be considered when interpreting the statistical analyses.

Finally, the present analysis reflects an early validation phase of haemodynamic simulation modelling prior to full-scale prospective calibration and iterative model refinement.

## 5. Conclusions

The present study provides an initial feasibility evaluation of a novel haemodynamic simulation software enabling patient-specific three-dimensional modelling of intracardiac shunt physiology based on routine echocardiographic data. Simulation-derived Qp:Qs quantification demonstrated no systematic difference from conventional Doppler-derived measurements, with small mean inter-method differences and no evidence of systematic bias.

Although predefined deviation thresholds were not fully achieved in this retrospective analysis, the modelling approach yielded haemodynamic estimates broadly comparable to established echocardiographic calculations while offering an integrated structural–functional assessment framework. These results should therefore be interpreted as proof-of-concept, and the software is not intended for clinical use at this stage. Processing was feasible within routine clinical workflows.

These findings support the feasibility of digital haemodynamic simulation as an emerging complementary modality for non-invasive shunt assessment. Ongoing prospective validation incorporating invasive haemodynamic measurements as well as multicentre registry data covering a broader range of cardiac morphologies and haemodynamic profiles will be essential to further define analytical accuracy, refine modelling performance, and determine the future clinical role of this technology.

## Figures and Tables

**Table 1:** Baseline demographic and echo data.

**Baseline table: ASD II cohort**			
Sex	Female	Number (%)	64 (65.3)
Age at echocardiography	Years	Median (min; max)	6 (0.6; 70)
BSA	m^2^	Median (min; max)	0.91 (0.28; 2.25)
Body weight	kg	Median (min; max)	23 (5; 100)
Body height	cm	Median (min; max)	121 (52; 184)
Type of closure	Heart catheter	Number (%)	79 (80.6)
	Surgery	Number (%)	19 (19.4)
**Echocardiographic parameter**			
ASD max./BSA	cm/m^2^	Mean (min; max)	0.85 (0.2; 2.25)
**Bicaval**			
ASD bicaval	cm/m^2^	Mean (min; max)	1.03 (0.2; 2.25)
IAS bicaval	cm/m^2^	Mean (min; max)	0.3 (0.13; 0.8)
SVC rim	cm/m^2^	Mean (min; max)	1.17 (0.37; 3.87)
IVC rim	cm/m^2^	Mean (min; max)	0.91 (0.24; 4.49)
**PSAX**			
ASD PSAX	cm/m^2^	Mean (min; max)	1 (0.26; 3.08)
IAS PSAX	cm/m^2^	Mean (min; max)	0.34 (0.13; 0.63)
Aortic rim	cm/m^2^	Mean (min; max)	2.02 (0.21; 17.44)
Atrial roof rim (PSAX)	cm/m^2^	Mean (min; max)	1.67 (0.26; 22.47)
**4CH**			
ASD 4CH	cm/m^2^	Mean (min; max)	0.94 (0.21; 2.77)
IAS 4CH	cm/m^2^	Mean (min; max)	0.27 (0.11; 0.64)
Crux cordis rim	cm/m^2^	Mean (min; max)	0.78 (0.26; 1.7)
Atrial roof rim (4CH)	cm/m^2^	Mean (min; max)	1.25 (0.28; 11.24)
**Atrial septal defect**			
Secundum ASD		Number (%)	98 (100)
Multiperforated ASD		Number (%)	0 (0)
Atrial septal aneurysm		Number (%)	4 (4.1)

Many patients underwent transcatheter closure (81%), while 19% received surgical repair. Patients treated surgically demonstrated significantly larger ASD diameters compared with those undergoing interventional closure. Mean ASD diameter in the surgical group was 19 mm (indexed: 24 mm/m^2^) versus 13 mm (indexed: 13 mm/m^2^) in the catheter group (*p* < 0.001).

## Data Availability

The data supporting the findings of this study are available upon request from the corresponding author. However, the data are not publicly accessible as they contain information that could compromise the privacy of the research participants.

## Abbreviations

The following abbreviations are used in this manuscript:

4CH	4-chamber view
ASD	Atrial septal defect
BSA	Body surface area
CI	Confidence interval
cMRI	Cardiovascular magnetic resonance imaging
CT	Computed tomography
DHZC	German Heart Center of the Charité (Deutsches Herzzentrum der Charité)
DICOM	Digital Imaging and Communications in Medicine
GDPR	General Data Protection Regulation
IAS	Interatrial septum
IVC	Inferior vena cava
PLAX	Parasternal long-axis view
PSAX	Parasternal short-axis view
Qp:Qs	Pulmonary-to-systemic blood flow ratio
Qp	Pulmonary blood flow
Qs	Systemic blood flow
REDCap	Research Electronic Data Capture
SPSS	Statistical Package for the Social Sciences
SVC	Superior vena cava
TEE	Transoesophageal echocardiography
TTE	Transthoracic echocardiography
VTI	Velocity time integral
